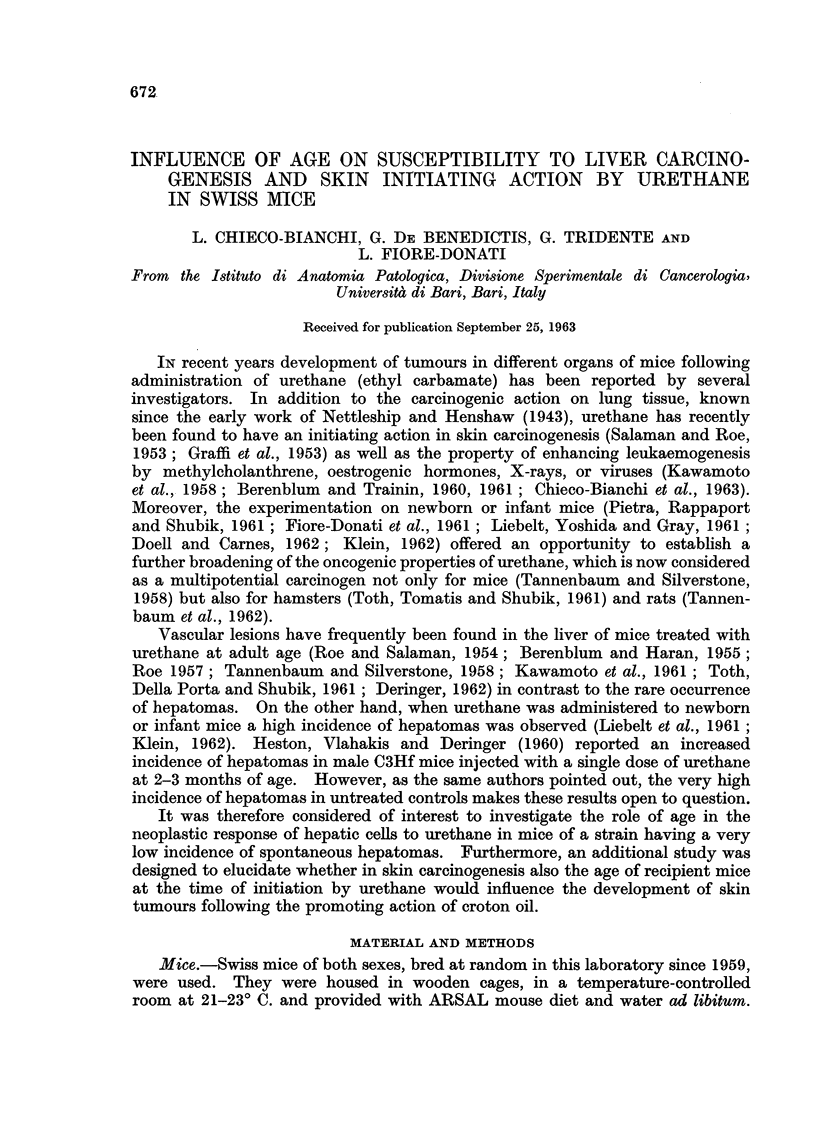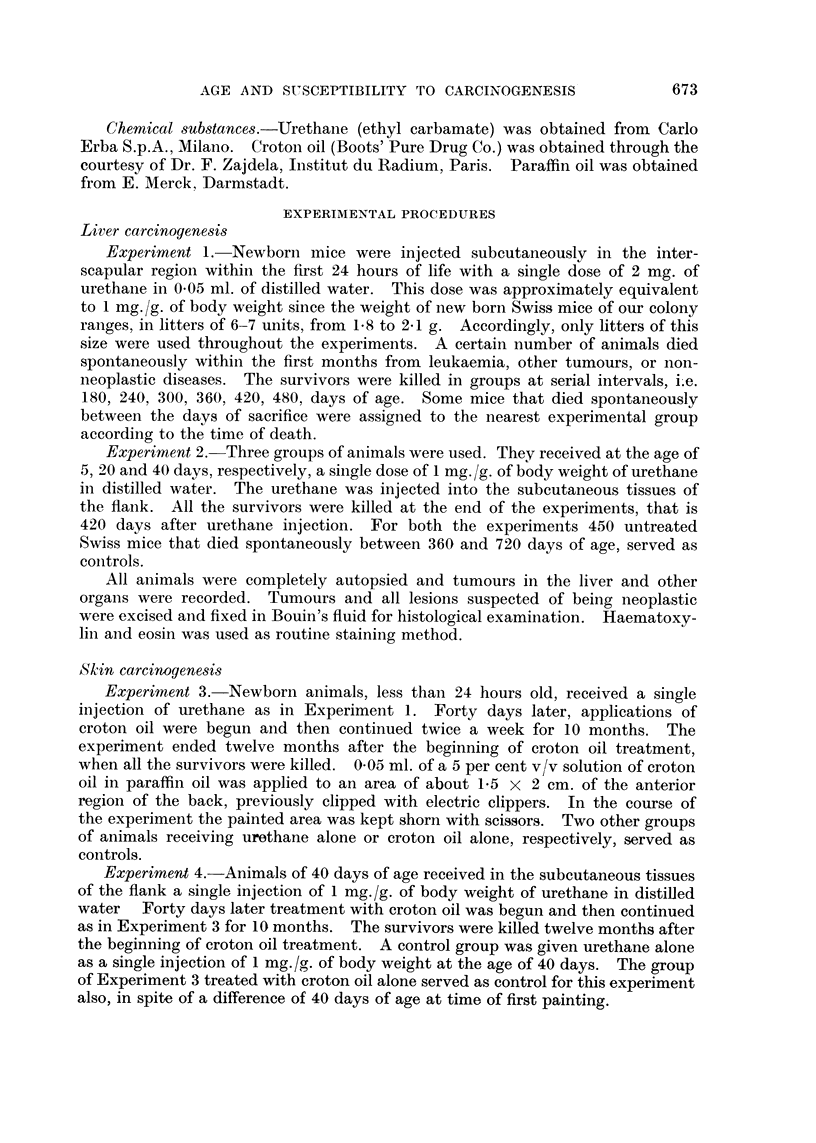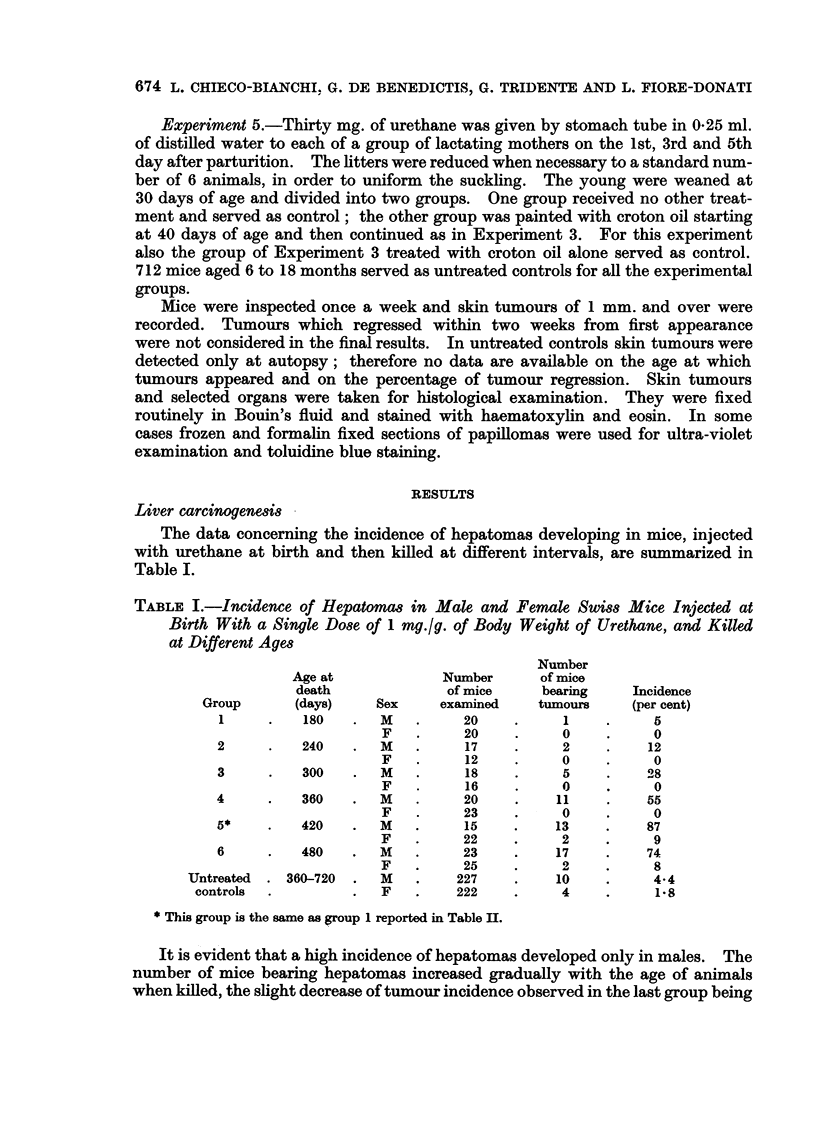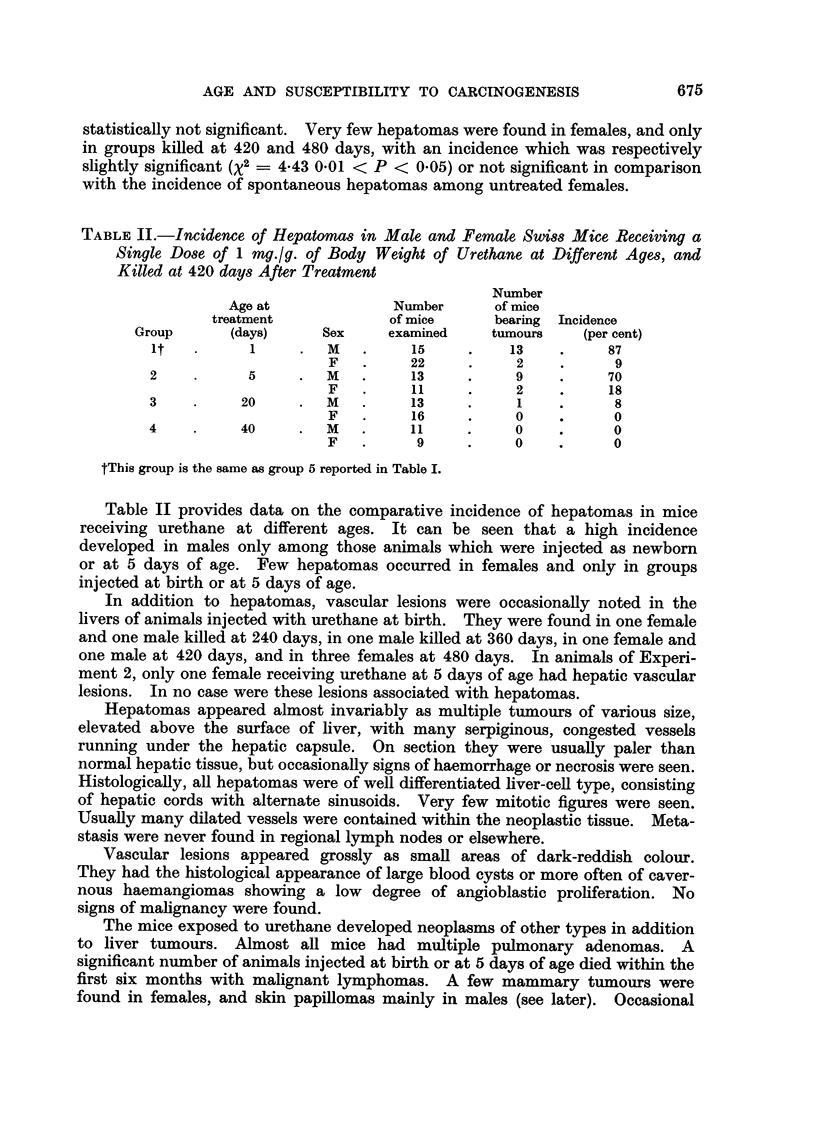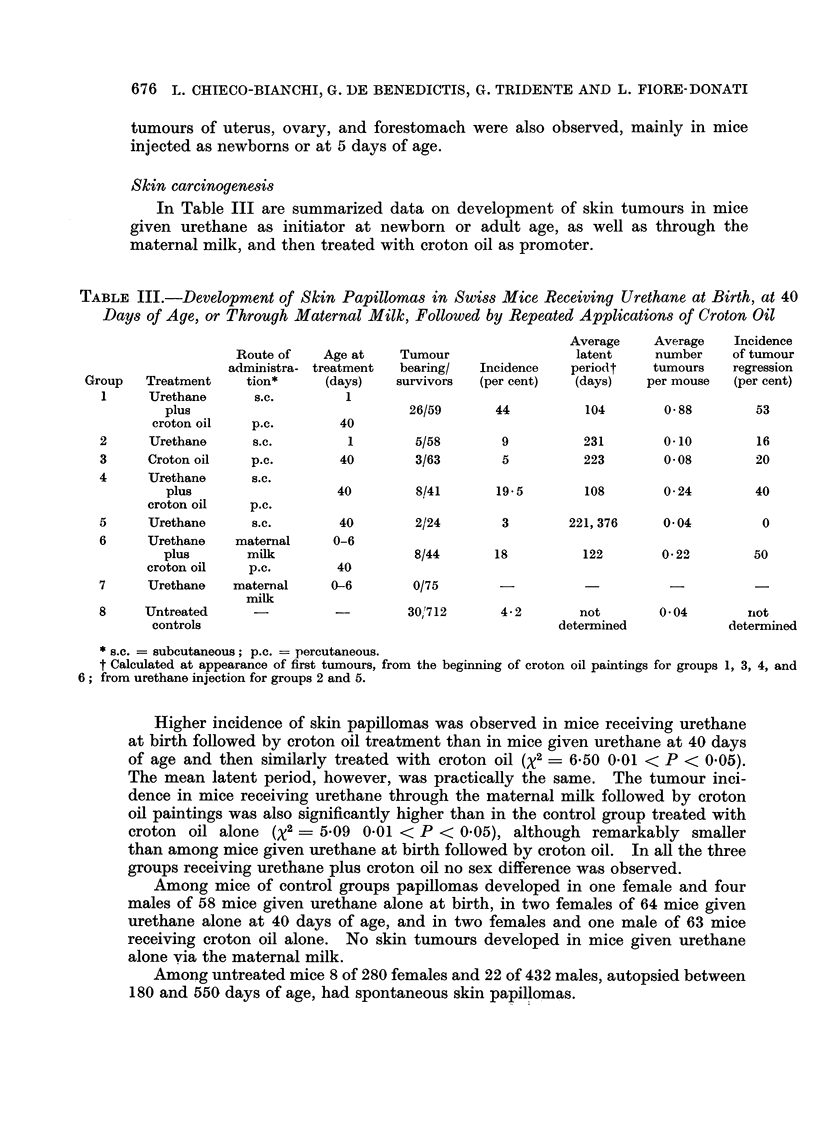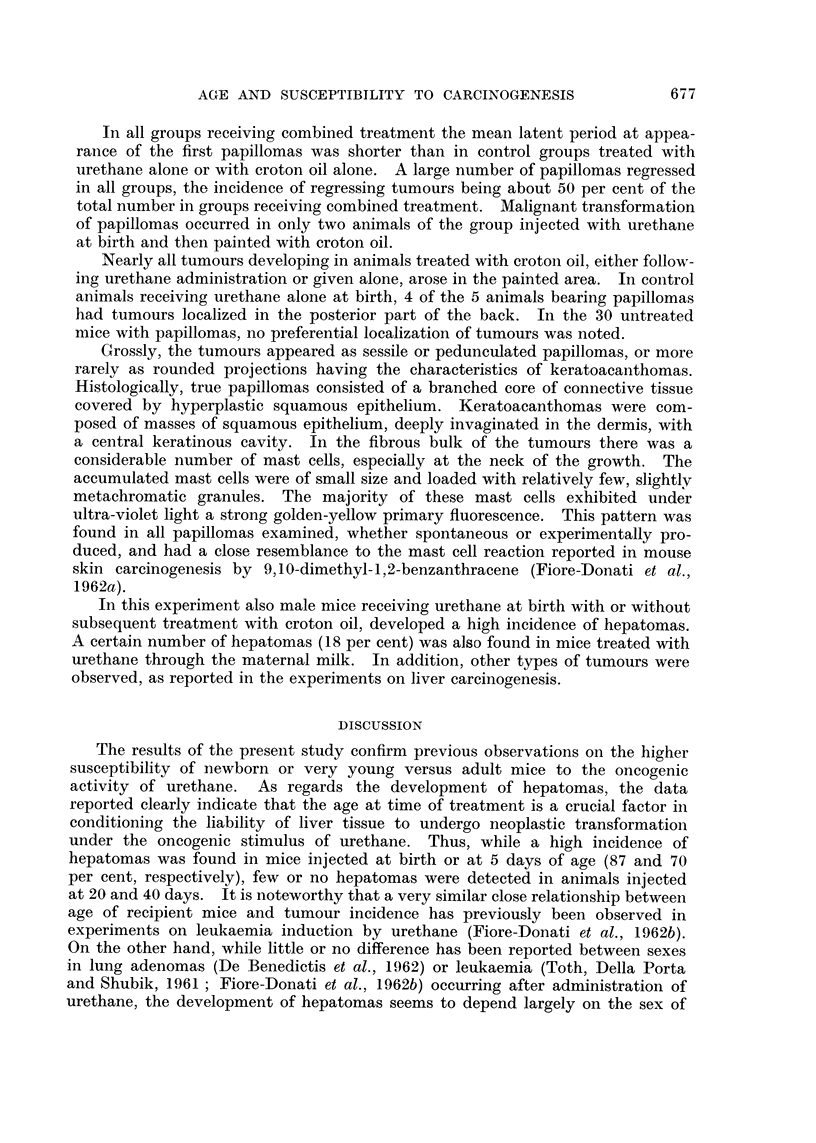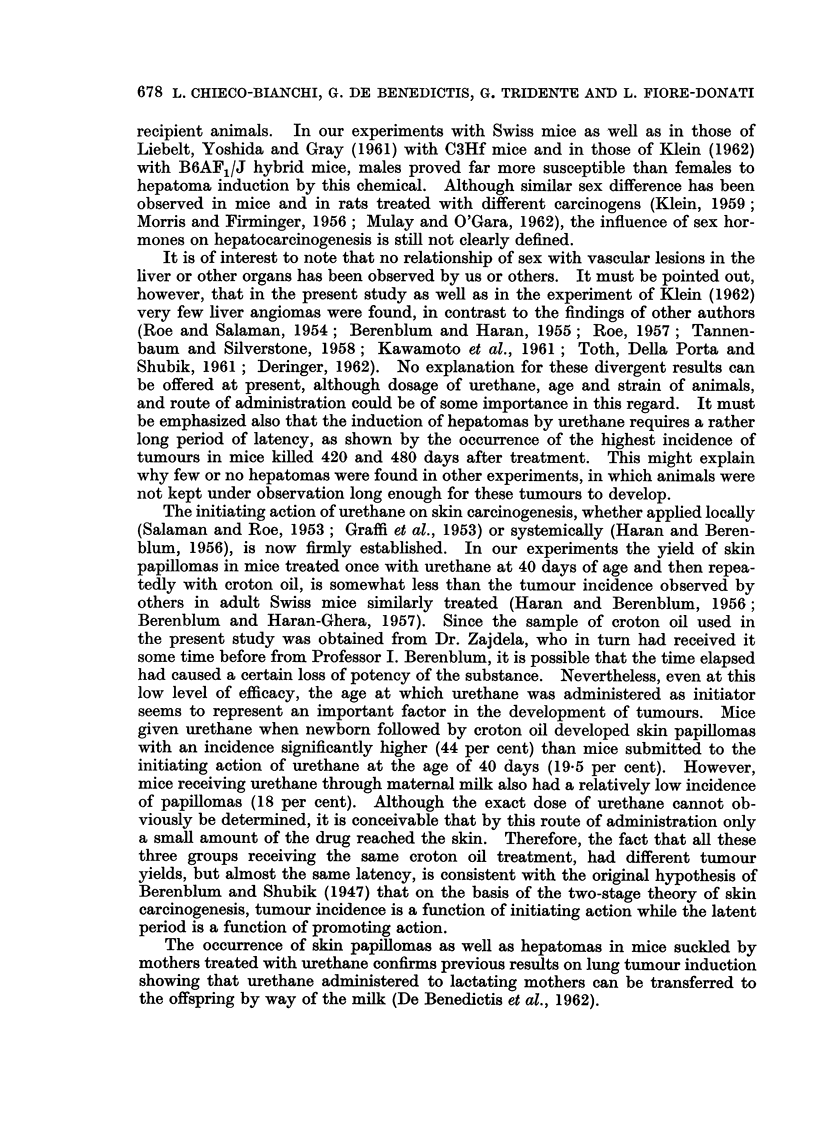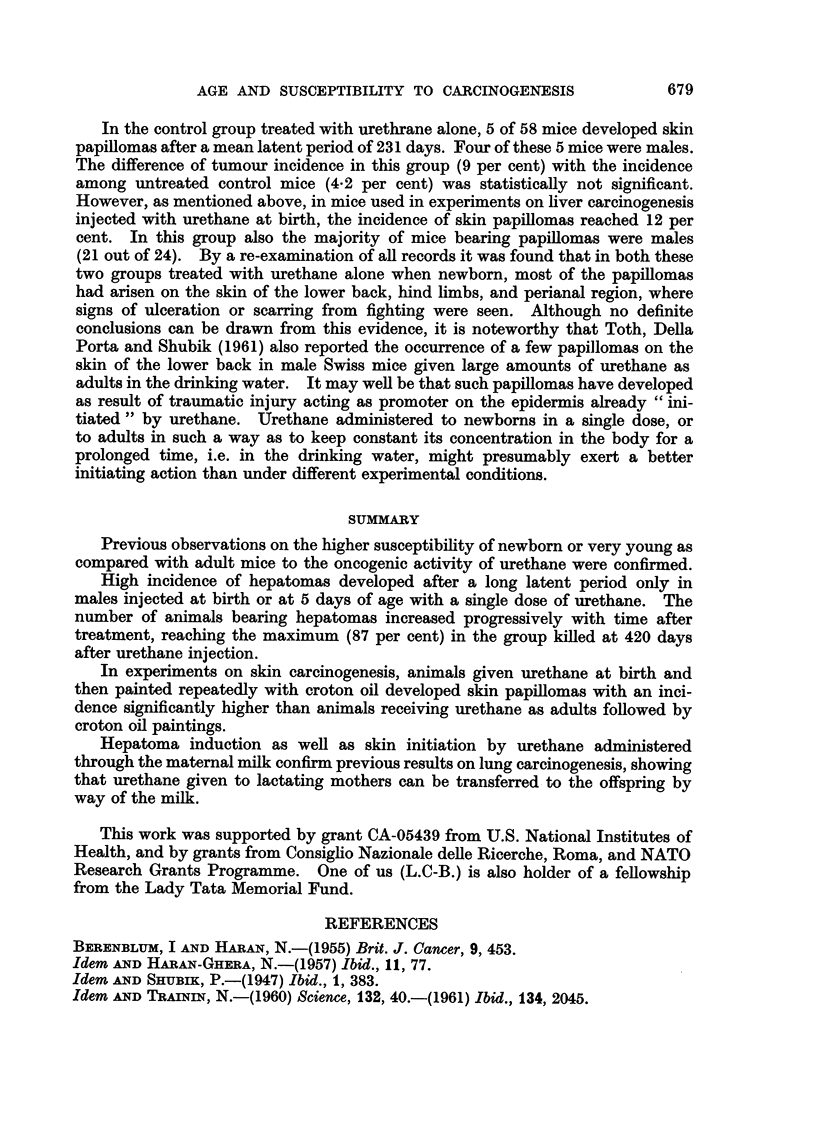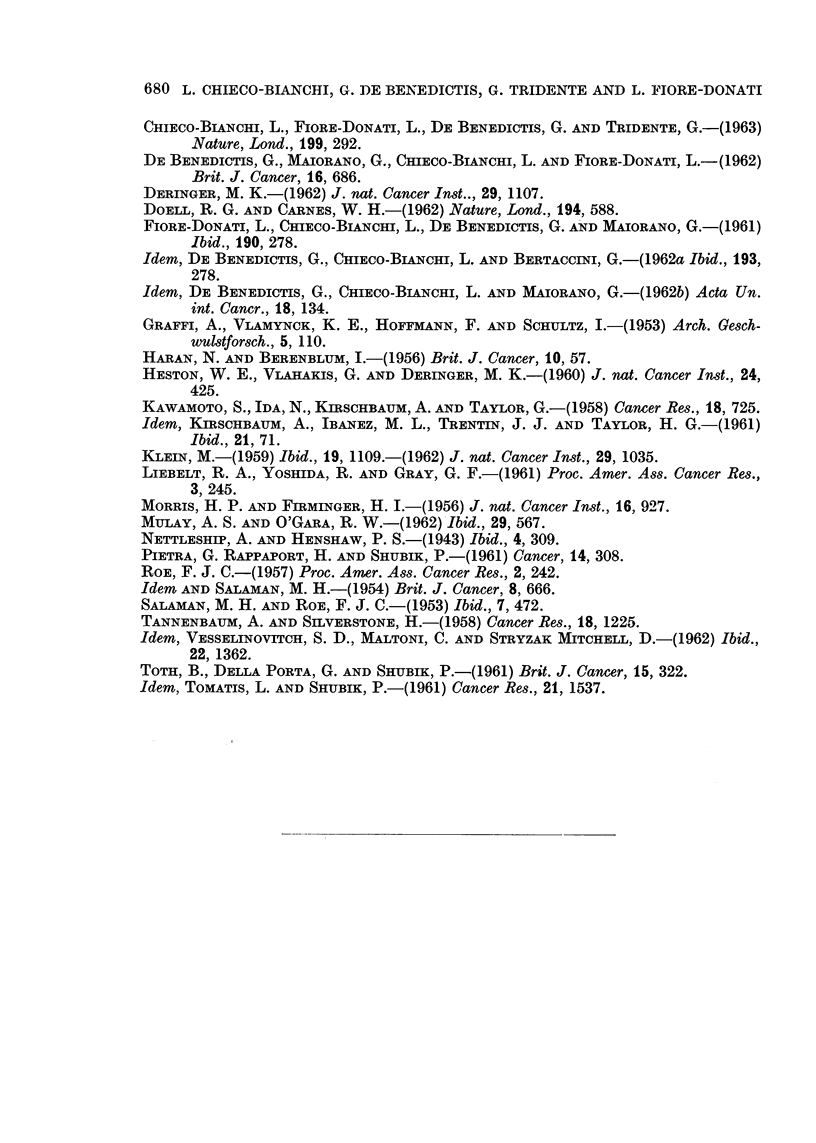# Influence of Age on Susceptibility to Liver Carcinogenesis and Skin Initiating Action by Urethane in Swiss Mice

**DOI:** 10.1038/bjc.1963.85

**Published:** 1963-12

**Authors:** L. Chieco-Bianchi, G. De Benedictis, G. Tridente, L. Fiore-Donati


### INFLUENCE OF AGE ON SUSCEPTIBILITY TO LIVER CARCINOGENESIS AND SKIN INITIATING ACTION BY URETHANE IN SWISS MICE

L. CHIECO-BIANCHI, G. DE BENEDICTIS, G. TRIDENTE AND L. FIORE-DONATI

*From the Istituto di Anatomia Patologica, Divisione Sperimentale di Cancerologia, Università di Bari, Bari, Italy*

Received for publication September 25, 1963

IN recent years development of tumours in different organs of mice following administration of urethane (ethyl carbamate) has been reported by several investigators. In addition to the carcinogenic action on lung tissue, known since the early work of Nettleship and Henshaw (1943), urethane has recently been found to have an initiating action in skin carcinogenesis (Salaman and Roe, 1953 ; Graffi *et al.*, 1953) as well as the property of enhancing leukaemogenesis by methylcholanthrene, oestrogenic hormones, X-rays, or viruses (Kawamoto *et al.*, 1958 ; Berenblum and Trainin, 1960, 1961 ; Chieco-Bianchi *et al.*, 1963). Moreover, the experimentation on newborn or infant mice (Pietra, Rappaport and Shubik, 1961 ; Fiore-Donati *et al.*, 1961 ; Liebelt, Yoshida and Gray, 1961 ; Doell and Carnes, 1962 ; Klein, 1962) offered an opportunity to establish a further broadening of the oncogenic properties of urethane, which is now considered as a multipotential carcinogen not only for mice (Tannenbaum and Silverstone, 1958) but also for hamsters (Toth, Tomatis and Shubik, 1961) and rats (Tannenbaum *et al.*, 1962).

Vascular lesions have frequently been found in the liver of mice treated with urethane at adult age (Roe and Salaman, 1954 ; Berenblum and Haran, 1955 ; Roe 1957 ; Tannenbaum and Silverstone, 1958 ; Kawamoto *et al.*, 1961 ; Toth, Della Porta and Shubik, 1961 ; Deringer, 1962) in contrast to the rare occurrence of hepatomas. On the other hand, when urethane was administered to newborn or infant mice a high incidence of hepatomas was observed (Liebelt *et al.*, 1961 ; Klein, 1962). Heston, Vlahakis and Deringer (1960) reported an increased incidence of hepatomas in male C3Hf mice injected with a single dose of urethane at 2–3 months of age. However, as the same authors pointed out, the very high incidence of hepatomas in untreated controls makes these results open to question.

It was therefore considered of interest to investigate the role of age in the neoplastic response of hepatic cells to urethane in mice of a strain having a very low incidence of spontaneous hepatomas. Furthermore, an additional study was designed to elucidate whether in skin carcinogenesis also the age of recipient mice at the time of initiation by urethane would influence the development of skin tumours following the promoting action of croton oil.

### MATERIAL AND METHODS

*Mice.*—Swiss mice of both sexes, bred at random in this laboratory since 1959, were used. They were housed in wooden cages, in a temperature-controlled room at 21–23° C. and provided with ARSAL mouse diet and water *ad libitum.*

*Chemical substances.*—Urethane (ethyl carbamate) was obtained from Carlo Erba S.p.A., Milano. Croton oil (Boots' Pure Drug Co.) was obtained through the courtesy of Dr. F. Zajdela, Institut du Radium, Paris. Paraffin oil was obtained from E. Merck, Darmstadt.

### EXPERIMENTAL PROCEDURES

### *Liver carcinogenesis*

*Experiment* 1.—Newborn mice were injected subcutaneously in the interscapular region within the first 24 hours of life with a single dose of 2 mg. of urethane in 0·05 ml. of distilled water. This dose was approximately equivalent to 1 mg./g. of body weight since the weight of new born Swiss mice of our colony ranges, in litters of 6–7 units, from 1·8 to 2·1 g. Accordingly, only litters of this size were used throughout the experiments. A certain number of animals died spontaneously within the first months from leukaemia, other tumours, or non-neoplastic diseases. The survivors were killed in groups at serial intervals, i.e. 180, 240, 300, 360, 420, 480, days of age. Some mice that died spontaneously between the days of sacrifice were assigned to the nearest experimental group according to the time of death.

*Experiment* 2.—Three groups of animals were used. They received at the age of 5, 20 and 40 days, respectively, a single dose of 1 mg./g. of body weight of urethane in distilled water. The urethane was injected into the subcutaneous tissues of the flank. All the survivors were killed at the end of the experiments, that is 420 days after urethane injection. For both the experiments 450 untreated Swiss mice that died spontaneously between 360 and 720 days of age, served as controls.

All animals were completely autopsied and tumours in the liver and other organs were recorded. Tumours and all lesions suspected of being neoplastic were excised and fixed in Bouin's fluid for histological examination. Haematoxylin and eosin was used as routine staining method.

### *Skin carcinogenesis*

*Experiment* 3.—Newborn animals, less than 24 hours old, received a single injection of urethane as in Experiment 1. Forty days later, applications of croton oil were begun and then continued twice a week for 10 months. The experiment ended twelve months after the beginning of croton oil treatment, when all the survivors were killed. 0·05 ml. of a 5 per cent v/v solution of croton oil in paraffin oil was applied to an area of about 1·5 × 2 cm. of the anterior region of the back, previously clipped with electric clippers. In the course of the experiment the painted area was kept shorn with scissors. Two other groups of animals receiving urethane alone or croton oil alone, respectively, served as controls.

*Experiment* 4.—Animals of 40 days of age received in the subcutaneous tissues of the flank a single injection of 1 mg./g. of body weight of urethane in distilled water Forty days later treatment with croton oil was begun and then continued as in Experiment 3 for 10 months. The survivors were killed twelve months after the beginning of croton oil treatment. A control group was given urethane alone as a single injection of 1 mg./g. of body weight at the age of 40 days. The group of Experiment 3 treated with croton oil alone served as control for this experiment also, in spite of a difference of 40 days of age at time of first painting.

*Experiment* 5.—Thirty mg. of urethane was given by stomach tube in 0·25 ml. of distilled water to each of a group of lactating mothers on the 1st, 3rd and 5th day after parturition. The litters were reduced when necessary to a standard number of 6 animals, in order to uniform the suckling. The young were weaned at 30 days of age and divided into two groups. One group received no other treatment and served as control ; the other group was painted with croton oil starting at 40 days of age and then continued as in Experiment 3. For this experiment also the group of Experiment 3 treated with croton oil alone served as control. 712 mice aged 6 to 18 months served as untreated controls for all the experimental groups.

Mice were inspected once a week and skin tumours of 1 mm. and over were recorded. Tumours which regressed within two weeks from first appearance were not considered in the final results. In untreated controls skin tumours were detected only at autopsy ; therefore no data are available on the age at which tumours appeared and on the percentage of tumour regression. Skin tumours and selected organs were taken for histological examination. They were fixed routinely in Bouin's fluid and stained with haematoxylin and eosin. In some cases frozen and formalin fixed sections of papillomas were used for ultra-violet examination and toluidine blue staining.

### RESULTS

*Liver carcinogenesis*

The data concerning the incidence of hepatomas developing in mice, injected with urethane at birth and then killed at different intervals, are summarized in Table I.

TABLE I.—*Incidence of Hepatomas in Male and Female Swiss Mice Injected at Birth With a Single Dose of* 1 *mg./g. of Body Weight of Urethane, and Killed at Different Ages*

| Group | Age at death (days) | Sex | Number of mice examined | Number of mice bearing tumours | Incidence (per cent) |
|---|---|---|---|---|---|
| 1 | 180 | M | 20 | 1 | 5 |
| | | F | 20 | 0 | 0 |
| 2 | 240 | M | 17 | 2 | 12 |
| | | F | 12 | 0 | 0 |
| 3 | 300 | M | 18 | 5 | 28 |
| | | F | 16 | 0 | 0 |
| 4 | 360 | M | 20 | 11 | 55 |
| | | F | 23 | 0 | 0 |
| 5* | 420 | M | 15 | 13 | 87 |
| | | F | 22 | 2 | 9 |
| 6 | 480 | M | 23 | 17 | 74 |
| | | F | 25 | 2 | 8 |
| Untreated | 360–720 | M | 227 | 10 | 4·4 |
| controls | | F | 222 | 4 | 1·8 |

* This group is the same as group 1 reported in Table II.

It is evident that a high incidence of hepatomas developed only in males. The number of mice bearing hepatomas increased gradually with the age of animals when killed, the slight decrease of tumour incidence observed in the last group being statistically not significant. Very few hepatomas were found in females, and only in groups killed at 420 and 480 days, with an incidence which was respectively slightly significant ($\chi^2 = 4{\cdot}43\ 0{\cdot}01 < P < 0{\cdot}05$) or not significant in comparison with the incidence of spontaneous hepatomas among untreated females.

TABLE II.—*Incidence of Hepatomas in Male and Female Swiss Mice Receiving a Single Dose of* 1 *mg./g. of Body Weight of Urethane at Different Ages, and Killed at* 420 *days After Treatment*

| Group | Age at treatment (days) | Sex | Number of mice examined | Number of mice bearing tumours | Incidence (per cent) |
|---|---|---|---|---|---|
| 1† | 1 | M | 15 | 13 | 87 |
| | | F | 22 | 2 | 9 |
| 2 | 5 | M | 13 | 9 | 70 |
| | | F | 11 | 2 | 18 |
| 3 | 20 | M | 13 | 1 | 8 |
| | | F | 16 | 0 | 0 |
| 4 | 40 | M | 11 | 0 | 0 |
| | | F | 9 | 0 | 0 |

†This group is the same as group 5 reported in Table I.

Table II provides data on the comparative incidence of hepatomas in mice receiving urethane at different ages. It can be seen that a high incidence developed in males only among those animals which were injected as newborn or at 5 days of age. Few hepatomas occurred in females and only in groups injected at birth or at 5 days of age.

In addition to hepatomas, vascular lesions were occasionally noted in the livers of animals injected with urethane at birth. They were found in one female and one male killed at 240 days, in one male killed at 360 days, in one female and one male at 420 days, and in three females at 480 days. In animals of Experiment 2, only one female receiving urethane at 5 days of age had hepatic vascular lesions. In no case were these lesions associated with hepatomas.

Hepatomas appeared almost invariably as multiple tumours of various size, elevated above the surface of liver, with many serpiginous, congested vessels running under the hepatic capsule. On section they were usually paler than normal hepatic tissue, but occasionally signs of haemorrhage or necrosis were seen. Histologically, all hepatomas were of well differentiated liver-cell type, consisting of hepatic cords with alternate sinusoids. Very few mitotic figures were seen. Usually many dilated vessels were contained within the neoplastic tissue. Metastasis were never found in regional lymph nodes or elsewhere.

Vascular lesions appeared grossly as small areas of dark-reddish colour. They had the histological appearance of large blood cysts or more often of cavernous haemangiomas showing a low degree of angioblastic proliferation. No signs of malignancy were found.

The mice exposed to urethane developed neoplasms of other types in addition to liver tumours. Almost all mice had multiple pulmonary adenomas. A significant number of animals injected at birth or at 5 days of age died within the first six months with malignant lymphomas. A few mammary tumours were found in females, and skin papillomas mainly in males (see later). Occasional tumours of uterus, ovary, and forestomach were also observed, mainly in mice injected as newborns or at 5 days of age.

*Skin carcinogenesis*

In Table III are summarized data on development of skin tumours in mice given urethane as initiator at newborn or adult age, as well as through the maternal milk, and then treated with croton oil as promoter.

TABLE III.—*Development of Skin Papillomas in Swiss Mice Receiving Urethane at Birth, at 40 Days of Age, or Through Maternal Milk, Followed by Repeated Applications of Croton Oil*

| Group | Treatment | Route of administration* | Age at treatment (days) | Tumour bearing/ survivors | Incidence (per cent) | Average latent period† (days) | Average number tumours per mouse | Incidence of tumour regression (per cent) |
|---|---|---|---|---|---|---|---|---|
| 1 | Urethane plus croton oil | s.c. p.c. | 1 40 | 26/59 | 44 | 104 | 0·88 | 53 |
| 2 | Urethane | s.c. | 1 | 5/58 | 9 | 231 | 0·10 | 16 |
| 3 | Croton oil | p.c. | 40 | 3/63 | 5 | 223 | 0·08 | 20 |
| 4 | Urethane plus croton oil | s.c. p.c. | 40 | 8/41 | 19·5 | 108 | 0·24 | 40 |
| 5 | Urethane | s.c. | 40 | 2/24 | 3 | 221, 376 | 0·04 | 0 |
| 6 | Urethane plus croton oil | maternal milk p.c. | 0–6 40 | 8/44 | 18 | 122 | 0·22 | 50 |
| 7 | Urethane | maternal milk | 0–6 | 0/75 | — | — | — | — |
| 8 | Untreated controls | — | — | 30/712 | 4·2 | not determined | 0·04 | not determined |

* s.c. = subcutaneous ; p.c. = percutaneous.
† Calculated at appearance of first tumours, from the beginning of croton oil paintings for groups 1, 3, 4, and 6 ; from urethane injection for groups 2 and 5.

Higher incidence of skin papillomas was observed in mice receiving urethane at birth followed by croton oil treatment than in mice given urethane at 40 days of age and then similarly treated with croton oil ($\chi^2 = 6{\cdot}50$ $0{\cdot}01 < P < 0{\cdot}05$). The mean latent period, however, was practically the same. The tumour incidence in mice receiving urethane through the maternal milk followed by croton oil paintings was also significantly higher than in the control group treated with croton oil alone ($\chi^2 = 5{\cdot}09$ $0{\cdot}01 < P < 0{\cdot}05$), although remarkably smaller than among mice given urethane at birth followed by croton oil. In all the three groups receiving urethane plus croton oil no sex difference was observed.

Among mice of control groups papillomas developed in one female and four males of 58 mice given urethane alone at birth, in two females of 64 mice given urethane alone at 40 days of age, and in two females and one male of 63 mice receiving croton oil alone. No skin tumours developed in mice given urethane alone via the maternal milk.

Among untreated mice 8 of 280 females and 22 of 432 males, autopsied between 180 and 550 days of age, had spontaneous skin papillomas.

In all groups receiving combined treatment the mean latent period at appearance of the first papillomas was shorter than in control groups treated with urethane alone or with croton oil alone. A large number of papillomas regressed in all groups, the incidence of regressing tumours being about 50 per cent of the total number in groups receiving combined treatment. Malignant transformation of papillomas occurred in only two animals of the group injected with urethane at birth and then painted with croton oil.

Nearly all tumours developing in animals treated with croton oil, either following urethane administration or given alone, arose in the painted area. In control animals receiving urethane alone at birth, 4 of the 5 animals bearing papillomas had tumours localized in the posterior part of the back. In the 30 untreated mice with papillomas, no preferential localization of tumours was noted.

Grossly, the tumours appeared as sessile or pedunculated papillomas, or more rarely as rounded projections having the characteristics of keratoacanthomas. Histologically, true papillomas consisted of a branched core of connective tissue covered by hyperplastic squamous epithelium. Keratoacanthomas were composed of masses of squamous epithelium, deeply invaginated in the dermis, with a central keratinous cavity. In the fibrous bulk of the tumours there was a considerable number of mast cells, especially at the neck of the growth. The accumulated mast cells were of small size and loaded with relatively few, slightly metachromatic granules. The majority of these mast cells exhibited under ultra-violet light a strong golden-yellow primary fluorescence. This pattern was found in all papillomas examined, whether spontaneous or experimentally produced, and had a close resemblance to the mast cell reaction reported in mouse skin carcinogenesis by 9,10-dimethyl-1,2-benzanthracene (Fiore-Donati *et al.*, 1962*a*).

In this experiment also male mice receiving urethane at birth with or without subsequent treatment with croton oil, developed a high incidence of hepatomas. A certain number of hepatomas (18 per cent) was also found in mice treated with urethane through the maternal milk. In addition, other types of tumours were observed, as reported in the experiments on liver carcinogenesis.

### DISCUSSION

The results of the present study confirm previous observations on the higher susceptibility of newborn or very young versus adult mice to the oncogenic activity of urethane. As regards the development of hepatomas, the data reported clearly indicate that the age at time of treatment is a crucial factor in conditioning the liability of liver tissue to undergo neoplastic transformation under the oncogenic stimulus of urethane. Thus, while a high incidence of hepatomas was found in mice injected at birth or at 5 days of age (87 and 70 per cent, respectively), few or no hepatomas were detected in animals injected at 20 and 40 days. It is noteworthy that a very similar close relationship between age of recipient mice and tumour incidence has previously been observed in experiments on leukaemia induction by urethane (Fiore-Donati *et al.*, 1962*b*). On the other hand, while little or no difference has been reported between sexes in lung adenomas (De Benedictis *et al.*, 1962) or leukaemia (Toth, Della Porta and Shubik, 1961 ; Fiore-Donati *et al.*, 1962*b*) occurring after administration of urethane, the development of hepatomas seems to depend largely on the sex of recipient animals. In our experiments with Swiss mice as well as in those of Liebelt, Yoshida and Gray (1961) with C3Hf mice and in those of Klein (1962) with $B6AF_1/J$ hybrid mice, males proved far more susceptible than females to hepatoma induction by this chemical. Although similar sex difference has been observed in mice and in rats treated with different carcinogens (Klein, 1959 ; Morris and Firminger, 1956 ; Mulay and O'Gara, 1962), the influence of sex hormones on hepatocarcinogenesis is still not clearly defined.

It is of interest to note that no relationship of sex with vascular lesions in the liver or other organs has been observed by us or others. It must be pointed out, however, that in the present study as well as in the experiment of Klein (1962) very few liver angiomas were found, in contrast to the findings of other authors (Roe and Salaman, 1954 ; Berenblum and Haran, 1955 ; Roe, 1957 ; Tannenbaum and Silverstone, 1958 ; Kawamoto *et al.*, 1961 ; Toth, Della Porta and Shubik, 1961 ; Deringer, 1962). No explanation for these divergent results can be offered at present, although dosage of urethane, age and strain of animals, and route of administration could be of some importance in this regard. It must be emphasized also that the induction of hepatomas by urethane requires a rather long period of latency, as shown by the occurrence of the highest incidence of tumours in mice killed 420 and 480 days after treatment. This might explain why few or no hepatomas were found in other experiments, in which animals were not kept under observation long enough for these tumours to develop.

The initiating action of urethane on skin carcinogenesis, whether applied locally (Salaman and Roe, 1953 ; Graffi *et al.*, 1953) or systemically (Haran and Berenblum, 1956), is now firmly established. In our experiments the yield of skin papillomas in mice treated once with urethane at 40 days of age and then repeatedly with croton oil, is somewhat less than the tumour incidence observed by others in adult Swiss mice similarly treated (Haran and Berenblum, 1956 ; Berenblum and Haran-Ghera, 1957). Since the sample of croton oil used in the present study was obtained from Dr. Zajdela, who in turn had received it some time before from Professor I. Berenblum, it is possible that the time elapsed had caused a certain loss of potency of the substance. Nevertheless, even at this low level of efficacy, the age at which urethane was administered as initiator seems to represent an important factor in the development of tumours. Mice given urethane when newborn followed by croton oil developed skin papillomas with an incidence significantly higher (44 per cent) than mice submitted to the initiating action of urethane at the age of 40 days (19·5 per cent). However, mice receiving urethane through maternal milk also had a relatively low incidence of papillomas (18 per cent). Although the exact dose of urethane cannot obviously be determined, it is conceivable that by this route of administration only a small amount of the drug reached the skin. Therefore, the fact that all these three groups receiving the same croton oil treatment, had different tumour yields, but almost the same latency, is consistent with the original hypothesis of Berenblum and Shubik (1947) that on the basis of the two-stage theory of skin carcinogenesis, tumour incidence is a function of initiating action while the latent period is a function of promoting action.

The occurrence of skin papillomas as well as hepatomas in mice suckled by mothers treated with urethane confirms previous results on lung tumour induction showing that urethane administered to lactating mothers can be transferred to the offspring by way of the milk (De Benedictis *et al.*, 1962).

In the control group treated with urethrane alone, 5 of 58 mice developed skin papillomas after a mean latent period of 231 days. Four of these 5 mice were males. The difference of tumour incidence in this group (9 per cent) with the incidence among untreated control mice (4·2 per cent) was statistically not significant. However, as mentioned above, in mice used in experiments on liver carcinogenesis injected with urethane at birth, the incidence of skin papillomas reached 12 per cent. In this group also the majority of mice bearing papillomas were males (21 out of 24). By a re-examination of all records it was found that in both these two groups treated with urethane alone when newborn, most of the papillomas had arisen on the skin of the lower back, hind limbs, and perianal region, where signs of ulceration or scarring from fighting were seen. Although no definite conclusions can be drawn from this evidence, it is noteworthy that Toth, Della Porta and Shubik (1961) also reported the occurrence of a few papillomas on the skin of the lower back in male Swiss mice given large amounts of urethane as adults in the drinking water. It may well be that such papillomas have developed as result of traumatic injury acting as promoter on the epidermis already " initiated " by urethane. Urethane administered to newborns in a single dose, or to adults in such a way as to keep constant its concentration in the body for a prolonged time, i.e. in the drinking water, might presumably exert a better initiating action than under different experimental conditions.

### SUMMARY

Previous observations on the higher susceptibility of newborn or very young as compared with adult mice to the oncogenic activity of urethane were confirmed.

High incidence of hepatomas developed after a long latent period only in males injected at birth or at 5 days of age with a single dose of urethane. The number of animals bearing hepatomas increased progressively with time after treatment, reaching the maximum (87 per cent) in the group killed at 420 days after urethane injection.

In experiments on skin carcinogenesis, animals given urethane at birth and then painted repeatedly with croton oil developed skin papillomas with an incidence significantly higher than animals receiving urethane as adults followed by croton oil paintings.

Hepatoma induction as well as skin initiation by urethane administered through the maternal milk confirm previous results on lung carcinogenesis, showing that urethane given to lactating mothers can be transferred to the offspring by way of the milk.

This work was supported by grant CA-05439 from U.S. National Institutes of Health, and by grants from Consiglio Nazionale delle Ricerche, Roma, and NATO Research Grants Programme. One of us (L.C-B.) is also holder of a fellowship from the Lady Tata Memorial Fund.

### REFERENCES

BERENBLUM, I AND HARAN, N.—(1955) *Brit. J. Cancer*, **9**, 453.

*Idem* AND HARAN-GHERA, N.—(1957) *Ibid.*, **11**, 77.

*Idem* AND SHUBIK, P.—(1947) *Ibid.*, **1**, 383.

*Idem* AND TRAININ, N.—(1960) *Science*, **132**, 40.—(1961) *Ibid.*, **134**, 2045.

CHIECO-BIANCHI, L., FIORE-DONATI, L., DE BENEDICTIS, G. AND TRIDENTE, G.—(1963) *Nature, Lond.*, **199**, 292.
DE BENEDICTIS, G., MAIORANO, G., CHIECO-BIANCHI, L. AND FIORE-DONATI, L.—(1962) *Brit. J. Cancer*, **16**, 686.
DERINGER, M. K.—(1962) *J. nat. Cancer Inst..*, **29**, 1107.
DOELL, R. G. AND CARNES, W. H.—(1962) *Nature, Lond.*, **194**, 588.
FIORE-DONATI, L., CHIECO-BIANCHI, L., DE BENEDICTIS, G. AND MAIORANO, G.—(1961) *Ibid.*, **190**, 278.
*Idem*, DE BENEDICTIS, G., CHIECO-BIANCHI, L. AND BERTACCINI, G.—(1962*a Ibid.*, **193**, 278.
*Idem*, DE BENEDICTIS, G., CHIECO-BIANCHI, L. AND MAIORANO, G.—(1962*b*) *Acta Un. int. Cancr.*, **18**, 134.
GRAFFI, A., VLAMYNCK, K. E., HOFFMANN, F. AND SCHULTZ, I.—(1953) *Arch. Geschwulstforsch.*, **5**, 110.
HARAN, N. AND BERENBLUM, I.—(1956) *Brit. J. Cancer*, **10**, 57.
HESTON, W. E., VLAHAKIS, G. AND DERINGER, M. K.—(1960) *J. nat. Cancer Inst.*, **24**, 425.
KAWAMOTO, S., IDA, N., KIRSCHBAUM, A. AND TAYLOR, G.—(1958) *Cancer Res.*, **18**, 725.
*Idem*, KIRSCHBAUM, A., IBANEZ, M. L., TRENTIN, J. J. AND TAYLOR, H. G.—(1961) *Ibid.*, **21**, 71.
KLEIN, M.—(1959) *Ibid.*, **19**, 1109.—(1962) *J. nat. Cancer Inst.*, **29**, 1035.
LIEBELT, R. A., YOSHIDA, R. AND GRAY, G. F.—(1961) *Proc. Amer. Ass. Cancer Res.*, **3**, 245.
MORRIS, H. P. AND FIRMINGER, H. I.—(1956) *J. nat. Cancer Inst.*, **16**, 927.
MULAY, A. S. AND O'GARA, R. W.—(1962) *Ibid.*, **29**, 567.
NETTLESHIP, A. AND HENSHAW, P. S.—(1943) *Ibid.*, **4**, 309.
PIETRA, G. RAPPAPORT, H. AND SHUBIK, P.—(1961) *Cancer*, **14**, 308.
ROE, F. J. C.—(1957) *Proc. Amer. Ass. Cancer Res.*, **2**, 242.
*Idem* AND SALAMAN, M. H.—(1954) *Brit. J. Cancer*, **8**, 666.
SALAMAN, M. H. AND ROE, F. J. C.—(1953) *Ibid.*, **7**, 472.
TANNENBAUM, A. AND SILVERSTONE, H.—(1958) *Cancer Res.*, **18**, 1225.
*Idem*, VESSELINOVITCH, S. D., MALTONI, C. AND STRYZAK MITCHELL, D.—(1962) *Ibid.*, **22**, 1362.
TOTH, B., DELLA PORTA, G. AND SHUBIK, P.—(1961) *Brit. J. Cancer*, **15**, 322.
*Idem*, TOMATIS, L. AND SHUBIK, P.—(1961) *Cancer Res.*, **21**, 1537.