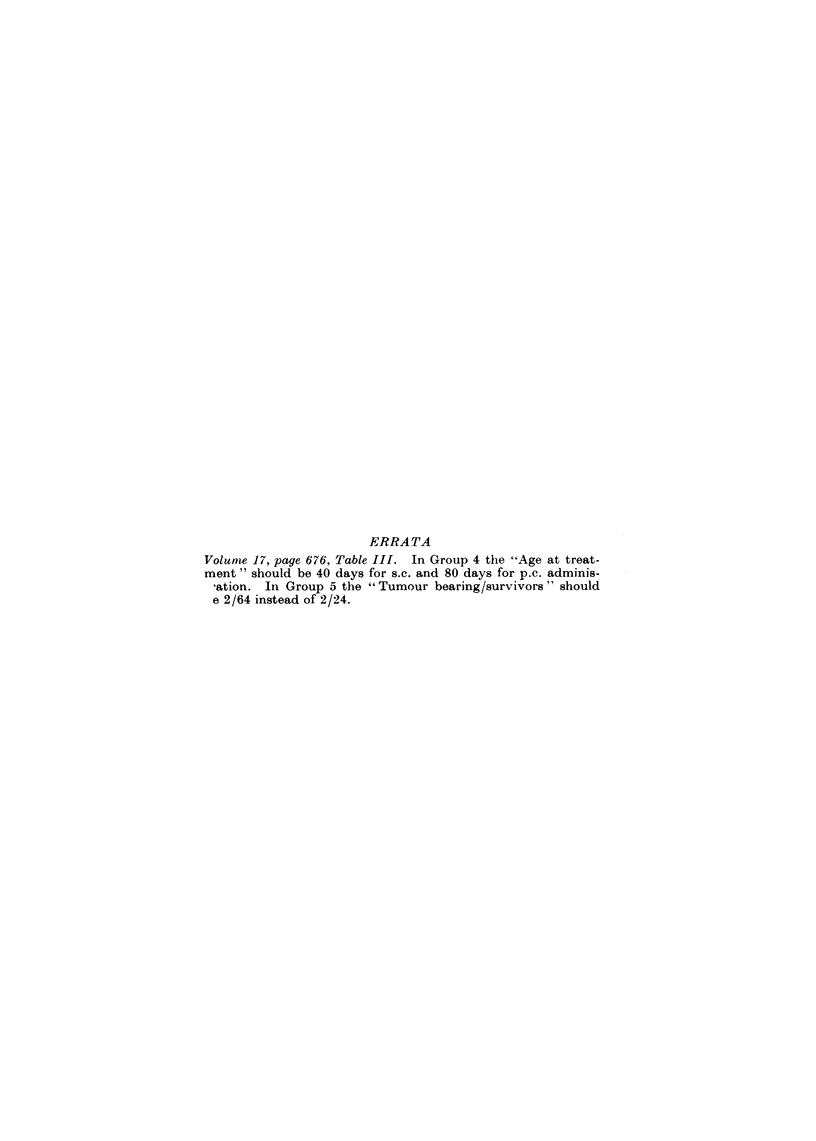# Errata

**Published:** 1964-06

**Authors:** 


### *ERRATA*

*Volume 17, page 676, Table III.* In Group 4 the "Age at treat-
ment" should be 40 days for s.c. and 80 days for p.c. adminis-
·ation. In Group 5 the "Tumour bearing/survivors" should
e 2/64 instead of 2/24.